# Identfication of viral and bacterial etiologic agents of the pertussis-like syndrome in children under 5 years old hospitalized

**DOI:** 10.1186/s12879-019-3671-6

**Published:** 2019-01-21

**Authors:** Stephanie Saiki-Macedo, Jorge Valverde-Ezeta, Angela Cornejo-Tapia, Maria Esther Castillo, Verónica Petrozzi-Helasvuo, Miguel Angel Aguilar-Luis, Luis J. del Valle, Erico Cieza-Mora, Carlos Bada, Olguita del Aguila, Wilmer Silva-Caso, Johanna Martins-Luna, Fernando Vasquez-Achaya, Juana del Valle-Mendoza

**Affiliations:** 1 School of Medicine. Research and Innovation Centre of the Faculty of Health Sciences, Universidad Peruana de Ciencias Aplicadas, Lima, Peru; 2 Instituto Nacional de Salud del Niño, Lima, Peru; 3 Facultad de Medicina, Hospital Nacional Cayetano Heredia, Lima, Peru; 4 Laboratorio de Biología Molecular, Instituto de Investigación Nutricional, Lima, Peru; 5 Instituto de Investigación de Enfermedades Infecciosas, Lima, Peru; 6 Barcelona Research Center for Multiscale Science and Engineering, Departament d’Enginyeria Química, EEBE, Universidad Politecnica de Catalunya (UPC), Barcelona Tech, Barcelona, Spain; 7 Hospita Regional Docente de Cajamarca, Cajamarca, Peru; 8 Hospital Nacional Edgardo Rebagliati Martins, Lima, Peru; 9 Hospital de Emergencias Pediátricas, Lima, Peru

**Keywords:** *Bordetella pertussis*, Acute respiratory infections, Adenovirus, *Mycoplasma pneumoniae*, Peru

## Abstract

**Background:**

Acute respiratory infections (ARIs) represent an important cause of morbidity and mortality in children, remaining a major public health concern, especially affecting children under 5 years old from low-income countries. Unfortunately, information regarding their epidemiology is still limited in Peru.

**Methods:**

A secondary data analysis was performed from a previous cross-sectional study conducted in children with a probable diagnosis of Pertussis from January 2010 to July 2012. All samples were analyzed via Polymerase Chain Reaction (PCR) for the following etiologies: Influenza-A, Influenza-B, RSV-A, RSV-B, Adenovirus, Parainfluenza 1 virus, Parainfluenza 2 virus, Parainfluenza 3 virus, *Mycoplasma pneumoniae* and *Chlamydia pneumoniae*.

**Results:**

A total of 288 patients were included. The most common pathogen isolated was Adenovirus (49%), followed by *Bordetella pertussis* (41%) from our previous investigation, the most prevelant microorganisms were *Mycoplasma pneumonia* (26%) and Influenza-B (19.8%). Coinfections were reported in 58% of samples and the most common association was found between *B. pertussis* and Adenovirus (12.2%).

**Conclusions:**

There was a high prevalence of Adenovirus, *Mycoplasma pneumoniae* and other etiologies in patients with a probable diagnosis of pertussis. Despite the presence of persistent cough lasting at least two weeks and other clinical characteristics highly suspicious of pertussis, secondary etiologies should be considered in children under 5 years-old in order to give a proper treatment.

**Electronic supplementary material:**

The online version of this article (10.1186/s12879-019-3671-6) contains supplementary material, which is available to authorized users.

## Background

Acute respiratory infections (ARIs) are a leading cause of morbidity, hospitalization, and mortality among children [1–3]. According to World Health Organization (WHO), acute respiratory infections are responsible for 1.9 million annual deaths in children, mainly affecting patients under 5 years old, with a higher incidence in those from low-income countries [1, 4].

ARIs are mainly caused by a wide range of viruses and bacteria [5, 6]. Viruses are isolated in up to 80% of cases, the most common pathogens are the respiratory syncytial virus (RSV) A and B, influenza (Flu) A, B and C, parainfluenza (PIV) types 1, 2, 3 and 4, coronavirus and rhinovirus [7, 8]. Classically, *S. pneumoniae* and *H. influenzae* type b are the most commonly isolated bacteria in both throat and nasopharyngeal specimens from patients with ARIs [9, 10]. However, in resource-limited countries, atypical bacteria such as *Mycoplasma pneumoniae*, *Chlamydia pneumoniae*, and *Bordetella pertussis* can play an important role in ARIs and can be detected in more than 40% of patients [2, 11–14].

Although numerous pathogens are associated with ARIs, their clinical manifestations are very similar, regardless of the causative agent. Thus, laboratory identification of the etiological agent is key in order to give a proper treatment and avoid the overuse of antibiotics [15]. Moreover, ARIs due to atypical bacterial infections have become a global concern especially after their reemergence in low-income countries [11, 16].

Simultaneous infections with virus and bacteria species have become an obstacle for clinicians, their prevalence has significantly increased, with studies discovering co-infections in more than 45% of cases [11, 17–19]. Additionally, these coinfections have been associated with longer hospitalization periods, worse clinical outcomes and increased mortality, again highlighting the importance of molecular etiological confirmation [17, 19, 20].

*Bordetella pertussis* represents a persistent cause of morbidity and mortality in children [21]. Accounting for an estimated 16 million cases and 195,000 deaths worldwide [22]. In a previous study we conducted on children under 1-year-old with a probable diagnosis of Pertussis from 5 Peruvian hospitals, we reported a prevalence of 39.54% pertussis cases [14]. With more than 60% of cases without an identified pathogen, hence a more comprehensive etiological analysis was required.

The main objective of this study was to detect the presence of 8 respiratory viruses (*Influenza-A, Influenza-B, RSV-A, RSV-B, Adenovirus, Parainfluenza-1, Parainfluenza-2 and Parainfluenza-3*) and atypical bacteria (*Mycoplasma pneumoniae, Chlamydia pneumonia*), via Polymerase Chain Reaction in samples from Peruvian children under 5 years-old previously analyzed for *B. Pertussis*.

## Methods

### Patients and study design

A secondary data analysis was performed from a previous cross-sectional study conducted from January 2010 to July 2012 in 5 Peruvian hospitals: Instituto Nacional de Salud Del Niño, Hospital Edgardo Rebagliati Martin, Hospital de Emergencias Pediátricas, Hospital Nacional Cayetano Heredia and Hospital Regional de Cajamarca.

**Fig. 1:**
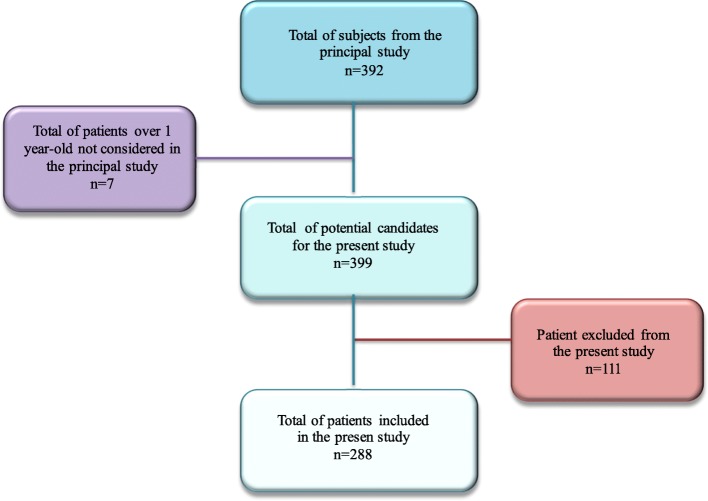
Flow chart with all patients enrolled in the study

The original study enrolled children under 5 years old admitted to the pediatric wards as probable Pertussis cases. However, in this original study, only patients under 1-year-old were included in the final analysis. A probable case was defined if patients presented paroxysms of coughing, or inspiratory “whoop” or post-tussive vomiting in the setting of an acute cough illness of any duration in the absence of another more likely diagnosis. In patients under 1 year-old apnea (with or without cyanosis) was also considered. All patients with chronic pulmonary conditions, cardiac disease or immunodeficiency were excluded. Patient who received antibiotics 7 days prior to the enrollement were also excluded. (Fig. 1).

This study included a total of 288 children under 5 years old hospitalized with a probable diagnosis of Pertussis, requiring further comprehensive etiological identification. All samples were analyzed via PCR for the following etiologies: Influenza-A, Influenza-B, RSV-A, RSV-B, Adenovirus, Parainfluenza 1 virus, Parainfluenza 2 virus, Parainfluenza 3 virus, *Mycoplasma pneumoniae* and *Chlamydia pneumoniae*.

### Ethics statement

This study has been approved by Ethics Committees of the *Universidad Peruana de Ciencias Aplicadas*. Parents and caregivers signed a written consent in the previous study which included a section that granted the investigators permission for a possible future use of the samples that could be given as an extension of the original research.

### Samples

Nasopharyngeal samples were obtained by inserting a swab into both nostrils parallel to the palate (Mini-Tip Culture Direct, Becton-Dickinson Microbiology System, MD 21152, USA) and a second swab from the posterior pharyngeal and tonsillar areas (Viral Culturette, Becton-Dickinson Microbiology Systems, MD, USA). Both nasal and pharyngeal swabs were placed into the same tube containing viral transport medium (minimal essential medium with 2% fetal bovine serum, amphotericin B 20 μg/ml, neomycin 40 μg/ml). Two aliquots of each fresh specimen were stored at − 20 °C to be used for posterior analysis of respiratory viruses and detect atypical pathogens by PCR.

### RNA/DNA extraction

RNA/DNA extraction was performed from 200 μL of the serum samples with the High Pure RNA Isolation Kit (Roche Applied Science, Mannheim, Germany), in accordance to the manufacturer’s instructions. Viral RNA/DNA obtained after extraction was eluted in 100 μl of nuclease-free water and then processed or stored at − 20 °C until use.

### Reverse transcription polymerase chain reaction (RT-PCR) for the analysis of respiratory viruses

RT-PCR for Influenza-A, Influenza-B, RSV-A, RSV-B and Adenovirus virus were performed using a BHQ quencher probe at 125 nM and 250 nM of primers in a final volume of 20 μl. Five microliters of the extracted RNA was combined with 15 μl of the master mix. RT-PCR conditions was 60 cycles of 15 s at 95 °C and 45 s at 60 °C. This process was performed in Light Cycler® 2.0 Instrument and the data was analyzed with the LightCycler® Software 4.1 (Roche Diagnostic, Deutschland-Mannheim, Germany). The primers and the probe for Influenza A and B were described by Selvaraju et al., 2010 [23], for RSV-A and RSV-B were described by Liu W. et al., 2016 [24] and for Adenovirus was described by Heim et al., 2003 [25].

For he case of Parainfluenza 1 virus, Parainfluenza 2 virus and Parainfluenza 3, the primers and conditions for RT-PCR were described by Coiras et al., 004 [26].

### Polymerase chain reaction (PCR) for the analysis of atypical pathogens *Mycoplasma pneumoniae*, *Chlamydia pneumoniae* and *Bordetella pertussis*

Polymerase chain reaction was performed with 5 μL of template DNA, polymerase (GoTaq; Promega, Madison, Wisconsin, USA). The Primers and conditios used for *Mycoplasma pneumoniae*, *Chlamydia pneumoniae* were descrited by del Valle et al.; 2017 [11] and primers and conditions used for *Bordetella pertussis* were describe by Castillo et al., 2015 [14]. Amplified products were recovered from the gel, purified (SpinPrep Gel DNA Kit; San Diego, CA) and sent for commercial sequencing (Macrogen, Korea).

### Statistical analysis

A database was generated in Microsoft Excel® 2015 (Microsoft Corporation, California, USA), all data was then exported to STATA® v13.0 (StataCorp, College Station, Texas, USA). Quantitative variables were described as frequencies and percentages for each group. The association was established by the Pearson correlation analysis (r) and the results presented in a scatter matrix with the ellipse plotted at 95% confidence.

## Results

A total of 288 patients under 5 years old with a probable diagnosis of *B. ertussis* were studied thouroughly for specific etiological identification. More than 80% of our study population were infants between 1 to 5 months old with a slightly higher number of males (56.3%). The group of infants between 29 days – 2 months-old (27.4%) and the group between 3 and 5 months-old (27.4%) were the most predominant, closely followed by the group between 2 and 3 months-old (26.4%) (Additional file 1: Table S1).

From our previous study, 118 cases of *Bordetella pertussis* were confirmed via PCR, leaving potentially 59% of samples without etiological identification. Thus, all 288 were analyzed for the presence of Influenza-A (Flu-A), Influenza-B (Flu-B), RSV-A, RSV-B, Adenovirus (ADV), Parainfluenza 1 virus (PIV-1), Parainfluenza 2 virus (PIV-2), Parainfluenza 3 virus (PIV-3), *Mycoplasma pneumoniae* and *Chlamydia pneumoniae*. The most common pathogen isolated was Adenovirus at 49% (141/288), followed by *Bordetella pertussis* at 41% (118/288), *Mycoplasma pneumonia* at 26% (75/288) and Flu-B at 19.8% (57/288) (Additional file 1: Table S1-A). The identification of these infectious agents has made it possible to establish remarkably that coinfections were present at 58% (167/288) of patients. Thus, cases of infection due to a single infectious agent were 28.8% (80/288), and where the presence of ADV was 10.2% (25/80) and for *B. pertussis* was 9.8% (24/80), followed by *M. pneumoniae* with 6.1% (15/80). Furthermore, the prevalence of these infectious agents were accumulated in children under 5 months of age (Additional file 1: Table S1-B).

As indicated above, in infected children (247 cases) coinfections stand out considerably (Additional file 2: Table S2). The coinfections found involve 2 to 6 different infectious agents, being the most frequent the coinfections of 2 agents with 39.6% (97/247) and those involving 3 agents with frequency of 23.2% (57/247). The most frequent association was the bacterial-viral coinfection, and the combination between *Bordetella-*ADV and *Mycoplasma-*ADV were the most common involvement reported with 12.2% (30/247) y 6.5% (16/247), respectively. However, the *Bordetella-Mycoplasma* association was very reduced (Additional file 2: Table S2), In addition, it is interesting to note that the associations in coinfections increase the frequency of infectious agents such as *Chlamydia*, Flu-B and RSV-A as observed in Additional file 1: Table S1 (compare 1A with 1B).

Regarding vaccination status, 65.25% (77/118) of the positive cases were unvaccinated. However, the majority of these children (40/77) were under two months of age. An unknown vaccinated status was observed in 5.93% (7/118) of patients positive for *B. pertussis*. A marked decrease was observed in children who had received at least one dose of vaccination, with a prevalence of 18.64% (22/118) (Additional file 3: Table S3).

**Fig. 2:**
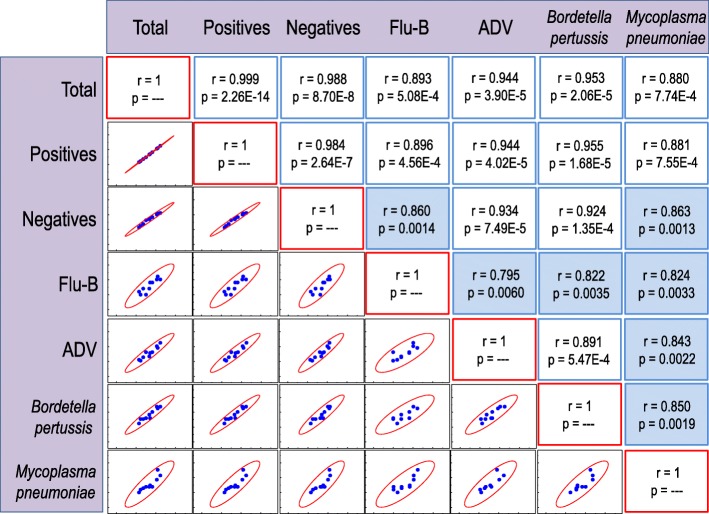
Association of the clinical symptoms in hospitalized children with a diagnostic for a single infectious agent. The graph is symmetrical on the diagonal. The squares in blue show the non-association

In our population, the most common clinical symptoms registered at admission were vomiting (47.2%), whooping (46.5%) and shortness of breath (43.1%), followed by fever (35.4%) and cyanosis (28.8%). A wide spread distribution of symptoms distribution was observed when patients symptoms were individually assessed based on etiological group. Only 6 pathogens had symptoms that were present in more than 50% of each group. For example, vomiting was more commonly reported among children with Flu-A, RSV-A, Parainfluenza-1 and *B. pertussis* (Additional file 4: Table S4-A)*.* However, the difficulty in establishing clear clinical symptoms associated with infectious causal agents is due to the high frequency of coinfections. Therefore, Additional file 4: Table S4-B has recorded the clinical symptoms of cases of infection with a single agent, and the association of these clinical symptoms are shown in Fig. 2. Thus, a clear non-association can be observed between Mycoplasma and Flu-B, ADV or Bordetella; the same happens for Flu-B and Bordetella or ADV. This non-association means that the only infectious agent could be identified taking into account the clinical symptoms of the children as shown in Additional file 4: Table S4-B.

The most common complications were acute bronchial obstructive syndrome (ABOS) and pneumonia in 56.6 and 28.1% of our population respectively. ABOS was the most frequent complications among patients with positive samples for RSV-A, Flu-A, ADV, *M. pneumoniae*, *C. pneumoniae* and *B. pertussis*. (Additional file 5: Table S5-A). However, when the complications of children affected by a single infectious agent are analyzed, it is clearly demonstrated that ABOS is also a complication of Flu-B. In addition, it is noteworthy that ABOS occurs in 61% (25/41) of the negative cases (Additional file 5: Table S5-B).

**Fig. 3:**
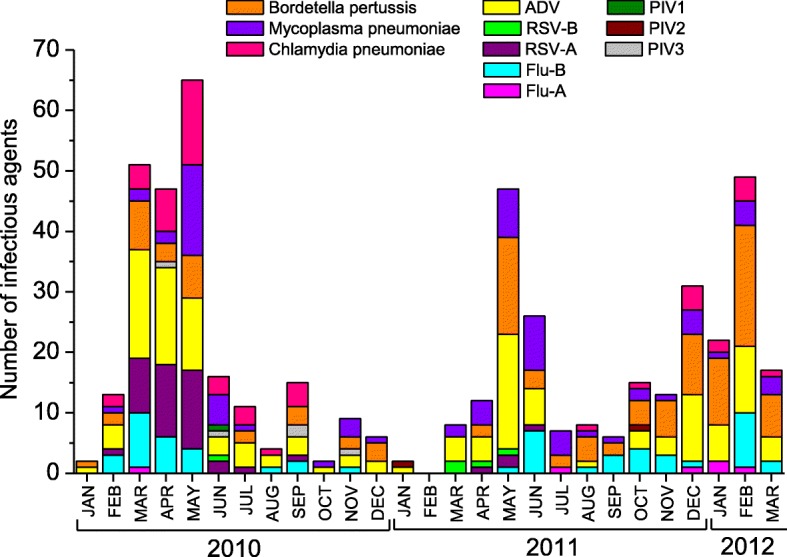
Viral and bacterial etiologies seasonal distribution

Finally, a seasonal distribution was described for each specific microorganism. Positive samples for ADV and *Mycoplasma pneumoniae* were observed across the whole study period. On the contrary, most of the *B. pertussis* cases were detected from May 2011 to March 2012. RSV-A and *Chlamydia* were mostly detected from March to May 2010; however, the same distribution was not observed in the following year (Fig. 3).

## Discussion

ARIs as the most common cause of morbidity and mortality in children, remaining a major concern, especially affecting children under 5 years old from low-income countries [1–4]. Unfortunately, information regarding their epidemiology is still limited in Peru [7, 11].

In recent years, there has been evidence of pertussis resurgence in Latin America, despite the introduction of the vaccine [14, 27, 28]. This bacterium is highly contagious and virulent, about half of the infected children under one year of whooping cough require hospitalization. In Peru, the national immunization program administers the combined pentavalent vaccine (DPT, HvB, Hib) at 2, 4, 6 months with reinforcements at 18 months and 4 years [29, 30]. Thus, we conducted a previous study on Peruvian children with a probable diagnosis of Pertussis and reported a *Bordetella pertussis* prevalence of 39.5% [14]. However, the classical presentation of pertussis has proven to be not enough to achieve a definitive diagnosis and laboratory tests are of the utmost importance for etiological confirmation to avoid overdiagnosis [28, 31, 32]. In the light of possible coinfections and more than 60% of patients without an etiological identification in our previous study, we conducted a new comprehensive analysis to detect viral and atypical bacterial etiologies in all our patients.

From a total of 288 samples analyzed from children under 5 years old with a probable diagnosis of Pertussis, the most common pathogen isolated was ADV in 49% of samples, followed by *B. pertussis* in 41% from our previous analysis. Although this study was conducted in patients with ARI with a highly suspicious pertussis diagnosis, other studies on children with ARI have identified ADV as one of the most prevalent etiologies [2] Although, the viral and bacterial prevalence may vary widely depending on the population characteristics [8, 33, 34].

Interestingly, our population were children with a highly suspicious clinical diagnosis of pertussis and despite that whooping was present in 46.5% of patients, ADV was the most common etiology isolated. Furthermore, a great number of patients with ADV infection presented with clinical symptoms very common among patients with pertussis such as whooping (51.1%), shortness of breath (43.4%) and vomiting (47.5%). Similarly, a recent study has reported that gastrointestinal symptoms and difficulty breathing are among the most common type of presentations in children [35]. Additionally, ADV has been historically identified as a major cause of pertussis-like syndrome, which results in the likelihood of a pertussis misdiagnosis in the absence of laboratory confirmation [36–38]. In this way, we have shown that patients with infection by ADV and *B.pertussis* as a single infectious agent have similar symptoms (Fig. 1).

It is also important to highlight the presence of *Mycoplasma pneumoniae* (26%) and *Chlamydia pneumoniae* (17.7%) among our patients. In a previous study, in children with ARIs, we reported a very similar prevalence of *Mycoplasma pneumoniae* and *Chlamydia pneumoniae* in 25.2 and 10.5%, respectively [11]. Demonstrating the high prevalence of these atypical bacteria among Peruvian children with ARIs. Our results from this current study also make noteworthy that clinical manifestations by *Mycoplasma pneumoniae* and Flu-B, ADV, or *B. pertussis* are distinguishable when the infection is due to infectious agent alone. However, in coinfections the symptoms were undestinguibles (see Table S4).

Additionally, in our previous study coinfections between these bacteria and viruses were also frequent; present in 67.4% of samples, coinfections between were the most common combination and the association between *Mycoplasma pneumoniae* with VRS-A was the most frequent one observed in 9.59% of patients [11]. Surprisingly, in this study, we have observed 58% of coinfections in our samples, again being the viral-bacterial association the most frequent and the most commonly detected coinfection involving *Bordetella pertussis-*ADV and *Mycoplasma pneumoniae*-ADV with frequencies of 12.2 and 6.5%, respectively. Another study in children, although in patients with community-acquired Pneumonia, also have reported coinfections in *M. pneumoniae* as the most common bacteria detected in association with a virus [39]. Thus, to avoid under-diagnosis, pertussis should be considered in patients with cough, especially if chronic, even when *M. pneumoniae* have been documented [40].

Another common coinfection was *B. pertussis* and Flu-B present in 9 patients. Although viral-bacterial coinfections are commonly associated with worse clinical courses and longer hospitalizations [17, 19, 20]. Recent investigations have reported similar clinical outcomes in infants hospitalized with *B. pertussis* and another respiratory virus coinfection [20]. However, noteworthy attention should be given to the *B. pertussis* and ADV coinfection in infants. A study compared infants with RSV and RSV-*B. pertussis* coinfection reporting similar disease severity; however, patients with this coinfection clearly needed more respiratory care and nutritional support [18]. Consequently, our only patient with RSV-A and pertussis presented with cyanosis and required advance respiratory support.

The variations in the rate and pattern of coinfection in patients with ARIs may be related to seasonal and geographical factors [39]. In our study, we intended to describe all detected pathogens and their seasonal distribution. Even though we were not able to describe any clear pattern, it is worth mentioning the high prevalence of ADV and *M. pneumoniae* across all of the study period, as well as the increasing prevalence of *B. Pertussis* on 2012.

## Limitations

Our study had some limitations. As mentioned in our previous study, due to our study design we were not able to establish causality between the pathogens isolated in our samples and our patient’s clinical presentation. Additionally, missing samples, we were not able to perform an etiological analysis in 104 samples from our previous study. We were not able to perform an etiological analysis in 104 samples from our previous study. Aditionally, there is still controversy if PCR alone can be used as a confirmatory method for *M. pneumoniae* diagnosis [41, 42]. Commercial PCR tests have high specificity and are currently a method of choice for direct pathogen detection [41, 43]. However, studies recommend that PCR alone should not replace serology and the combination of both could be good screening tests for reliable and accurate diagnosis of M. pneumoniae [41]. Moreover, other more recent studies suggest that a positive PCR or serology for M. pneumoniae may be unable to differentiate between asymptomatic carriage and symptomatic infection [44].

To date, the available published information regarding the etiological prevalence of ARIs in Peruvian children is still limited. Despite the high incidence of Pertussis, especially in vulnerable populations such as infants, to date to establish a etiological diagnosis in low income countries is still challenging [11, 16, 43]. Moreover, the relationship between the clinical severity and coinfections in respiratory pathogens remains inconclusive. Our study is among the first ones to describe multiple viral and bacterial etiologies in patients with a high clinical suspicion of pertussis [45, 46]. Further investigations should be conducted in order to understand the role of these pathogens in Peruvian children.

## Additional files

Additional file 1: **Table S1.** Demographics in children with a probable diagnosis of Pertussis, positives for respiratory virus and atypical bacteria. (DOCX 141 kb)

Additional file 2: **Table S2.** Clinical symptoms in hospitalized children with a probable diagnosis of Pertussis, positives for respiratory virus and atypical bacteria. (DOCX 19 kb)

Additional file 3: **Table S3.** Vaccination status in *B. pertussis*-positive patients (DOCX 43 kb)

Additional file 4: **Table S4.** Coinfections in hospitalized children with a probable diagnosis of Pertussis, positives for respiratory virus and atypical bacteria. (DOCX 142 kb)

Additional file 5: **Table S5.** Complications among hospitalized children with a probable diagnosis of Pertussis, positives for respiratory virus and atypical bacteria. (DOCX 123 kb)

## Abbreviations

ARIs: Acute respiratory infections
DNA: Deoxyribonucleic acid
FLU: influenza
PCR: *polymerase chain reaction*
PIV: Parainfluenza
RNA: Ribonucleica cid; bp: base pair
RSV: Respiratory syncytial virus

## References

[1] Williams B, Gouws E, Boschi-Pinto C, Bryce J, Dye C (2002). Estimates of world-wide distribution of child deaths from acute respiratory infections. Lancet Infect Dis.

[2] Assane D, Makhtar C, Abdoulaye D, Amary F, Djibril B, Amadou D (2018). Viral and Bacterial Etiologies of Acute Respiratory Infections Among Children Under 5 Years in Senegal. Microbiol Insights.

[3] Selvaraj K, Chinnakali P, Majumdar A, Krishnan I (2014). Acute respiratory infections among under-5 children in India: a situational analysis. J Nat Sci Biol Med.

[4] Mulholland K (2003). Global burden of acute respiratory infections in children: implications for interventions. Pediatr Pulmonol.

[5] Rhedin S, Lindstrand A, Rotzén-Östlund M, Tolfvenstam T, Ohrmalm L, Rinder MR, Zweygberg-Wirgart B, Ortqvist A, Henriques-Normark B, Broliden K, Naucler P (2014). Clinical utility of PCR for common viruses in acute respiratory illness. Pediatrics.

[6] Althouse BM, Flasche S, Minh LN, Thiem VD, Hashizume M, Ariyoshi K, Anh DD, Rodgers GL, Klugman KP, Hu H, Yoshida LM. Seasonality of respiratory viruses causing hospitalizations for acute respiratory infections in children in Nha Trang, Vietnam. Int J Infect Dis. 2018;75:18-25. 10.1016/j.ijid.2018.08.001 PMC7110808 30118916

[7] Camps Serra M, Cervera C, Pumarola T, Moreno A, Perelló R, Torres A, Jiménez de Anta MT, Marcos MA. Virological diagnosis in community-acquired pneumonia in immunocompromised patients. Eur Respir J. 2008;31(3):618-24. 10.1183/09031936.00073807 17959637

[8] Fernandes-Matano L, Monroy-Muñoz I, Angeles-Martinez J, Sarquiz-Martinez B, Patomec-Nava I, Pardave-Alejandre H (2017). Prevalence of non-influenza respiratory viruses in acute respiratory infection cases in Mexico. PLoS One.

[9] Berman S (1991). Epidemiology of acute respiratory infections in children of developing countries. Rev Infect Dis.

[10] Mohammed E, Muhe L, Geyid A, Dejene A, Mekonnen Y, Mammo K (2000). Prevalence of bacterial pathogens in children with acute respiratory infection in Addis Ababa. Ethiop Med J.

[11] Del Valle-Mendoza J, Orellana-Peralta F, Marcelo-Rodriguez A, Verne E, Esquivel-Vizcarra M, Silva-Caso W (2017). High prevalence of mycoplasma pneumoniae and chlamydia pneumoniae in children with acute respiratory infections from Lima. Peru PLoS One.

[12] Del Valle-Mendoza J, Silva-Caso W, Cornejo-Tapia A, Orellana-Peralta F, Verne E, Ugarte C (2017). Molecular etiological profile of atypical bacterial pathogens, viruses and coinfections among infants and children with community-acquired pneumonia admitted to a national hospital in Lima. Peru BMC Res Notes.

[13] Al-ssum RM, Al-Malki MA. Use of multiplex PCR for diagnosis of bacterial infection respiratory mixed. Malaysian Journal of Microbiology. 2010;6(1) Available from: http://web.usm.my/mjm/issues/vol6no1/research1.pdf.

[14] Castillo ME, Bada C, Del Aguila O, Petrozzi-Helasvuo V, Casabona-Ore V, Reyes I, Del Valle-Mendoza J (2015). Detection of Bordetella pertussis using a PCR test in infants younger than one year old hospitalized with whooping cough in five Peruvian hospitals. Int J Infect Dis.

[15] Bhuyan G, Hossain M, Sarker S, Rahat A, Islam M, Hague T (2017). Bacterial and viral pathogen spectra of acute respiratory infections in under-5 children in hospital settings in Dhaka city. PLoS One.

[16] Syed M, Bana N (2014). Pertussis: a reemerging and an underreported infectious disease. Saudi Med J.

[17] Mina M, Burke R, Klugman K (2014). Estimating the prevalence of coinfection with influenza virus and the atypical bacteria Bordetella pertussis, Chlamydophila pneumoniae, and mycoplasma pneumoniae. Eur J Clin Microbiol Infect Dis.

[18] Moreno M, Amores M, Pradillo M, Moreno-Perez D, Cordon A, Urda A (2015). Incidence and severity of pertussis in infants with a respiratory syncytial virus infection [article in Spanish]. Enferm Infecc Microbiol Clin.

[19] Damasio G, Pereira L, Moreira S, Duarte C, Dalla-Costa L, Raboni S (2015). Does virus-bacteria coinfection increase the clinical severity of acute respiratory infection?. J Med Virol.

[20] Frassanito A, Nenna R, Nicolai A, Pierangeli A, Tozzi A, Stefanelli P (2017). Infants hospitalized for Bordetella pertussis infection commonly have respiratory viral coinfections. BMC Infect Dis.

[21] Cantey JB, Sánchez PJ, Tran J, Chung W, Siegel JD (2014). Pertussis: a persistent cause of morbidity and mortality in young infants. J Pediatr.

[22] WHO | Pertussis [Internet]. Who. int. 2017 [cited 9 February 2018]. Available from: http://www.who.int/immunization/en/.

[23] Selvaraju SB, Selvarangan R (2010). Evaluation of three influenza a and B real-time reverse transcription-PCR assays and a new 2009 H1N1 assay for detection of influenza viruses. J Clin Microbiol.

[24] Liu W, Chen D, Tan W, Xu D, Qiu S, Zeng Z, Li X, Zhou R (2016). Epidemiology and clinical presentations of respiratory syncytial virus subgroups a and B detected with multiplex real-time PCR. PLoS One.

[25] Heim A, Ebnet C, Harste G, Pring-Akerblom P (2003). Rapid and quantitative detection of human adenovirus DNA by real-time PCR. J Med Virol.

[26] Coiras MT, Aguilar JC, García ML, Casas I, Pérez-Breña P (2004). Simultaneous detection of fourteen respiratory viruses in clinical specimens by two multiplex reverse transcription nested-PCR assays. J Med Virol.

[27] Pan American Health Organization (PAHO). Alerta Epidemiológica: Tos Ferina (Coqueluche). [Internet]. Washington, D.C. [Accessed May 22, 2017; Cited on January 11, 2018] Available at: http://www.paho.org/hq/index.php?option=com_docman&task=doc_view&gid=19325&Itemid.

[28] Van den Brink G, Wishaupt J, Douma J (2014). Bordetella pertussis: an underreported pathogen in pediatric respiratory infections, a prospective cohort study. BMC Infect Dis.

[29] MINSA Perú. NTS N° 080 -MINSA/DGSP V.03 (2013 [Technical health standard for the national vaccination] http://www.minsa.gob.pe/diresahuanuco/ESRI/pdf/RM510_2013_MINSA_Esquema%0Nacional%20de%20Vacunaci%C3%B3n.pdf. Accessed on 15 Aug 2013.

[30] Centers for Disease Control and Prevention (2014) Childhood Whooping Cough Vaccine Protects Most Children For At Least 5 years. Available: http://www.cdc.gov/pertussis/surv-reporting.html. Accessed 7 Sept 2014.

[31] Matto S, Cherry J (2005). Molecular pathogenesis, epidemiology, and clinical manifestations of respiratory infections due to Bordetella pertussis and other Bordetella subspecies. Clin Microbiol Rev.

[32] Hajia M, Rahbar M, Fallah F (2012). Detection of Bordetella pertussis in infants suspected to have whooping cough. Open Respir Med J.

[33] Del Valle J, Cornejo-Tapia A, Weilg P, Verne E, Nazario-Fuertes R, Ugarte C (2015). Incidence of respiratory viruses in Peruvian children with acute respiratory infections. J Med Virol.

[34] Fillatre A, Francois C, Segard C, Duverlie G, Hecquet D, Pannier C (2018). Epidemiology and seasonality of acute respiratory infections in hospitalized children over four consecutive years (2012-2016). J Clin Virol.

[35] Jobran S, Kattan R, Shamaa J, Marzouga H, Hindiyeh M (2018). Adenovirus respiratory tract infections in infants: a retrospective chart-review study. Lancet.

[36] Sarbay H, Polat A, Mete E, Balci Y, Akin M (2016). Pertussis-like syndrome associated with adenovirus presenting with hyperleukocytosis: case report. North Clin Istanb.

[37] Vargosko A (1970). Whooping-cough due to adenovirus. Br Med J.

[38] Nelson K, Gavitt F, Batt M, Kallick C, Reddi K, Levin S (1975). The role of adenoviruses in the pertussis syndrome. J Pediatr.

[39] Jiang W, Wu M, Zhou J, Wang Y, Hao C, Ji W (2017). Etiologic spectrum and occurrence of coinfections in children hospitalized with community-acquired pneumonia. BMC Infect Dis.

[40] Cheon M, Na H, Han S, Kwon H, Chun Y, Kang J (2015). Pertussis accompanying recent mycoplasma infection in a 10-year-old girl. Infect Chemother.

[41] Zhang L, Zong ZY, Liu YB, Ye H, Lv XJ (2011). PCR versus serology for diagnosing mycoplasma pneumoniae infection: a systematic review & meta-analysis. Indian J Med Res.

[42] Daxboeck F, Krause R, Wenisch C (2003). Laboratory diagnosis of mycoplasma pneumoniae infection. Clin Microbiol Infect.

[43] Masseria C, Marti C, Krishnarajh G, Becker L, Buikema A, Tan T (2017). Incidence and burden of pertussis among infants less than 1 year of age. Pediatr Infect Dis J.

[44] Spuesens EB, Fraaij PL, Visser EG (2013). Carriage of mycoplasma pneumoniae in the upper respiratory tract of symptomatic and asymptomatic children: an observational study. PLoS Med.

[45] Ferronato A, Gilio A, Vieira S (2013). Respiratory viral infections in infants with clinically suspected pertussis. J Pediatr.

[46] Nicolai A, Nenna R, Frassanito A, Pierangeli A (2016). Respiratory viruses and B pertussis co-infections: A frequent occurrence in children hospitalized with B pertussis. Eur Resp J.

