# Supplementary material for: Identfication of viral and bacterial etiologic agents of the pertussis-like syndrome in children under 5 years old hospitalized
Source: BMC Infect Dis. 2019 Jan 21;19:75. doi: 10.1186/s12879-019-3671-6 (PMC6341522; doi:10.1186/s12879-019-3671-6)
Supplement: Supplementary file 1 — Table S1. Demographics in children with a probable diagnosis of Pertussis, positives for respiratory virus and atypical bacteria. (DOCX 141 kb) [file 12879_2019_3671_MOESM1_ESM.docx]

**Table S1. Demographics in children with a probable diagnosis of Pertussis, positives for respiratory virus and atypical bacteria.**

| 1. **Frequencies of infection** | | | | | | | | | | | | |
| --- | --- | --- | --- | --- | --- | --- | --- | --- | --- | --- | --- | --- |
| **Age** | **N= 288 (%)** | **Flu-A** | **Flu-B** | **RSV-A** | **RSV-B** | **ADV** | **PIV-1** | **PIV-2** | **PIV-3** | ***Bordetella pertussis*** | ***Mycoplasma pneumoniae*** | ***Chlamydia pneumoniae*** |
|  |  | n=6 (%) | n=57(%) | n=43 (%) | n=5 (%) | n=141 (%) | n=5 (%) | n=2 (%) | n=2 (%) | n=118 (%) | n=75 (%) | n=51 (%) |
| ≤ 28 d | 17 (5.9) | --- | 2 (3.5) | 3 (7.0) | --- | 4 (2.8) | --- | --- | --- | 5 (4.2) | 7 (9.3) | 4 (7.8) |
| 29 d – < 2 month | 79 (27.4) | 2 (33.3) | 15 (26.3) | 14 (32.6) | 4 (80.0) | 42 (29.8) | 1 (20.0) | --- | --- | 35 (29.7) | 22 (29.3) | 16 (31.4) |
| 2 - < 3 month | 76 (26.4) | 1 (16.7) | 13 (22.8) | 11 (25.6) | 1 (20.0) | 37 (26.2) | --- | --- | 1 (50.0) | 36 (30.5) | 17 (22.7) | 13 (25.5) |
| 3 - 5 month | 79 (27.4) | 3 (50.0) | 16 (28.1) | 11 (25.6) | --- | 39 (27.7) | 4 (80.0) | 1 (50.0) | --- | 30 (25.4) | 19 (25.3) | 15 (29.4) |
| 6 - 11 month | 30 (10.4) | --- | 10 (17.5) | 3 (7.0) | --- | 16 (11.4) | --- | 1 (50.0) | --- | 10 (8.5) | 7 (9.3) | 3 (5.9) |
| 1 -5 year | 7 (2.4) | --- | 1 (1.8) | 1 (2.3) | --- | 3 (2.1) | --- | --- | 1 (50.0) | 2 (1.7) | 3 (4.0) | 0 (0.0) |
| **Gender** |  |  |  |  |  |  |  |  |  |  |  |  |
| Male | 162 (56.3) | 2 (33.3) | 25 (43.9) | 21 (48.8) | 3 (60.0) | 84 (59.6) | 2 (40.0) | 1 (50.0) | 1 (50.0) | 60 (50.9) | 45 (60.0) | 25 (49.0) |
| Female | 126 (43.8) | 4 (66.7) | 32 (56.1) | 22 (51.2) | 2 (40.0) | 57 (40.4) | 3 (60.0) | 1 (50.0) | 1 (50.0) | 58 (49.2) | 30 (40.0) | 26 (51.0) |
| 1. **Frequencies of infection with a single infectious agent in the positive-cases** | | | | | | | | | | | | |
| **Age** | **N= 80**  **(%)** | **Flu-A** | **Flu-B** | **RSV-A** | **RSV-B** | **ADV** | **PIV-1** | **PIV-2** | **PIV-3** | ***Bordetella pertussis*** | ***Mycoplasma pneumoniae*** | ***Chlamydia pneumoniae*** |
|  |  | n=0 (%) | n=7 (%) | n=4 (%) | n=0 (%) | n=25 (%) | n=1 (%) | n=1 (%) | n=1 (%) | n=24 (%) | n=15 (%) | n=2 (%) |
| ≤ 28 d | 4 (5.0) | --- | --- | 1 (25.0) | --- | --- | --- | --- | --- | 1 (4.2) | 2 (13.3) | --- |
| 29 d – < 2 month | 21 (26.2) | --- | 2 (28.6) | 1 (25.0) | --- | 7 (28.0) | 1 (100.0) | --- | --- | 5 (20.8) | 4 (26.7) | 1 (50.0) |
| 2 - < 3 month | 27 (33.8) | --- | 2 (28.6) | 1 (25.0) | --- | 6 (24.0) | --- | --- | 1 (100.0) | 12 (50.0) | 5 (33.3) | --- |
| 3 - 5 month | 17 (21.2) | --- | 1 (14.3) | 1 (25.0) | --- | 9 (36.0) | --- | --- | --- | 4 (16.7) | 1 (6.7) | 1 (50.0) |
| 6 - 11 month | 9 (11.2) | --- | 2 (28.6) | --- | --- | 2 (8.0) | --- | 1 (100.0) | --- | 1 (4.2) | 3 (2) | --- |
| 1 -5 year | 2 (2.5) | --- | --- | --- | --- | 1 (4.0) | --- | --- | --- | 1 (4.2) | --- | --- |
| **Gender** |  |  |  |  |  |  |  |  |  |  |  |  |
| Male | 54 (67.5) | --- | 6 (85.7) | 2 (50.0) | --- | 17 (68.0) | --- | --- | 1 (100.0) | 13 (54.2) | 13 (86.7) | 1 (50.0) |
| Female | 26 (32.5) | --- | 1 (14.3) | 2 (50.0) | --- | 8 (32.0) | 1 (100.0) | 1 (100.0) | --- | 11 (45.8) | 2 (13.3) | 1 (50.0) |
