# Supplementary material for: Identfication of viral and bacterial etiologic agents of the pertussis-like syndrome in children under 5 years old hospitalized
Source: BMC Infect Dis. 2019 Jan 21;19:75. doi: 10.1186/s12879-019-3671-6 (PMC6341522; doi:10.1186/s12879-019-3671-6)
Supplement: Supplementary file 2 — Table S2. Clinical symptoms in hospitalized children with a probable diagnosis of Pertussis, positives for respiratory virus and atypical bacteria. (DOCX 19 kb) [file 12879_2019_3671_MOESM2_ESM.docx]

**Table S2. Coinfections in hospitalized children with a probable diagnosis of Pertussis, positives for respiratory virus and atypical bacteria.**

| **_Etiologic Agents_** | | | **_N_**  **_(247)_** | **_%_** | **_Total_**  **_%_** |
| --- | --- | --- | --- | --- | --- |
| **_Number_** | **_Type_** | **_Species_** |  |  |  |
| _One-Infectious Agent_ | _Viral_ | _ADV_ | _25_ | _10.2_ |  |
|  |  | _Flu-B_ | _7_ | _2.9_ |  |
|  |  | _RSV-A_ | _4_ | _1.6_ |  |
|  |  | _PIV-1_ | _1_ | _0.4_ |  |
|  |  | _PIV-2_ | _1_ | _0.4_ |  |
|  |  | _PIV-3_ | _1_ | _0.4_ | _15.9_ |
|  | _Bacterial_ | *_B. pertussis_* | _24_ | _9.8_ |  |
|  |  | *_M. pneumoniae_* | _15_ | _6.1_ |  |
|  |  | *_C. pneumoniae_* | _2_ | _0.8_ | _16.7_ |
| _Two-Infectious Agents_ | _Viral_ | _ADV + Flu-B_ | _7_ | _2.9_ |  |
|  |  | _ADV + Flu-A_ | _1_ | _0.4_ |  |
|  |  | _ADV + RSV-A_ | _4_ | _1.6_ |  |
|  |  | _ADV + RSV-B_ | _2_ | _0.8_ |  |
|  |  | _ADV + PIV-1_ | _1_ | _0.4_ |  |
|  |  | _ADV + PIV-2_ | _1_ | _0.4_ |  |
|  |  | _RSV-A + Flu-B_ | _1_ | _0.4_ | _6.9_ |
|  | _Bacterial_ | *_B. pertussis_* ***_+_*** *_C. pneumoniae_* | _2_ | _0.8_ |  |
|  |  | *_B. pertussis + M. pneumoniae_* | _3_ | _1.2_ |  |
|  |  | *_C. pneumoniae + M. pneumoniae_* | _2_ | _0.8_ | _2.9_ |
|  | _Bacterial-Viral_ | *_B. pertussis_* **_+_** _ADV_ | _30_ | _12.2_ |  |
|  |  | *_B. pertussis_* _+ RSV-A_ | _1_ | _0.4_ |  |
|  |  | *_B. pertussis_* _+ Flu-B_ | _9_ | _3.7_ |  |
|  |  | *_C. pneumoniae_* _+ ADV_ | _4_ | _1.6_ |  |
|  |  | *_C. pneumoniae_* _+ RSV-A_ | _6_ | _2.4_ |  |
|  |  | *_C. pneumoniae_* _+ RSV-B_ | _1_ | _0.4_ |  |
|  |  | *_M. pneumoniae_* _+ ADV_ | _16_ | _6.5_ |  |
|  |  | *_M. pneumoniae_* _+ RSV-A_ | _3_ | _1.2_ |  |
|  |  | *_M. pneumoniae_* _+ Flu-B_ | _3_ | _1.2_ | _29.8_ |
| _Three-Infectious Agents_ | _Viral_ | _ADV + RSV-A + Flu-B_ | _4_ | _1.6_ | _1.6_ |
|  | _Bacterial_ | *_B. pertussis + C. pneumoniae + M. pneumoniae_* | _4_ | _1.6_ | _1.6_ |
|  | _Bacterial-Viral_ | *_B. pertussis_* _+ ADV + RSV-A_ | _3_ | _1.2_ |  |
|  |  | *_B. pertussis_* _+ ADV + Flu-B_ | _5_ | _2.0_ |  |
|  |  | *_B. pertussis_* _+ Flu-B + PIV-1_ | _1_ | _0.4_ |  |
|  |  | *_C. pneumoniae_* _+ ADV + RSV-A_ | _5_ | _2.0_ |  |
|  |  | *_M. pneumoniae_* _+ ADV + Flu-B_ | _3_ | _1.2_ |  |
|  |  | *_M. pneumoniae_* _+ ADV + RSV-A_ | _1_ | _0.4_ |  |
|  |  | *_M. pneumoniae_* _+ ADV + RSV-B_ | _1_ | _0.4_ |  |
|  |  | *_M. pneumoniae_* _+ Flu-B + RSV-A_ | _1_ | _0.4_ |  |
|  |  | *_B. pertussis + C. pneumoniae +_* _ADV_ | _7_ | _2.9_ |  |
|  |  | *_B. pertussis + C. pneumoniae_* _+ Flu-A_ | _1_ | _0.4_ |  |
|  |  | *_B. pertussis + C. pneumoniae_* _+ Flu-B_ | _3_ | _1.2_ |  |
|  |  | *_B. pertussis + C. pneumoniae_* _+ RSV-A_ | _1_ | _0.4_ |  |
|  |  | *_B. pertussis + C. pneumoniae_* _+ PIV-1_ | _1_ | _0.4_ |  |
|  |  | *_B. pertussis + M. pneumoniae_* _+ Flu-A_ | _1_ | _0.4_ |  |
|  |  | *_B. pertussis + M. pneumoniae_* _+ Flu-B_ | _3_ | _1.2_ |  |
|  |  | *_B. pertussis + M. pneumoniae_* _+ ADV_ | _8_ | _3.3_ |  |
|  |  | *_B. pertussis + M. pneumoniae_* _+ PIV-1_ | _1_ | _0.4_ |  |
|  |  | *_B. pertussis + M. pneumoniae_* _+ PIV-3_ | _1_ | _0.4_ |  |
|  |  | *_C. pneumoniae + M. pneumoniae_* _+ RSV-A_ | _2_ | _0.8_ | _20.0_ |
| _Four-Infectious Agents_ | _Bacterial-Viral_ | *_B. pertussis +_* _ADV + RSV-A + Flu-A_ | _1_ | _0.4_ |  |
|  |  | *_B. pertussis +_* _ADV + Flu-A + Flu-B_ | _1_ | _0.4_ |  |
|  |  | *_C. pneumoniae +_* _ADV + RSV-A + Flu-B_ | _2_ | _0.8_ |  |
|  |  | *_M. pneumoniae_* _+ ADV + RSV-A + Flu-B_ | _1_ | _0.4_ |  |
|  |  | *_B. pertussis + C. pneumoniae +_* _ADV + Flu-A_ | _1_ | _0.4_ |  |
|  |  | *_B. pertussis + M. pneumoniae +_* _ADV + Flu-B_ | _1_ | _0.4_ |  |
|  |  | *_C. pneumoniae + M. pneumoniae_* _+ ADV + RSV-A_ | _1_ | _0.4_ |  |
|  |  | *_B. pertussis + C. pneumoniae + M. pneumoniae +_* _RSV-A_ | _1_ | _0.4_ | _3.7_ |
| _Five-_  _Infectious Agents_ | _Bacterial-Viral_ | *_B. pertussis + C. pneumoniae + M. pneumoniae_*  _+ ADV + Flu-B_ | _1_ | _0.4_ | _0.4_ |
| _Six-Infectious Agents_ | _Bacterial-Viral_ | *_B. pertussis + C. pneumoniae + M. pneumoniae_*  *_+_* _ADV + RSV-A + Flu-B_ | _1_ | _0.4_ | _0.4_ |
