# Supplementary material for: Identfication of viral and bacterial etiologic agents of the pertussis-like syndrome in children under 5 years old hospitalized
Source: BMC Infect Dis. 2019 Jan 21;19:75. doi: 10.1186/s12879-019-3671-6 (PMC6341522; doi:10.1186/s12879-019-3671-6)
Supplement: Supplementary file 3 — Table S3. Vaccination status in B. pertussis-positive patients (DOCX 43 kb) [file 12879_2019_3671_MOESM3_ESM.docx]

**Table S3. Vaccination status in *B. pertussis*-positive patients**

| **DPT (doses)** | **Total patients with DPT vaccine** | | **Patients *B. pertusis* positive by PCR** | |
| --- | --- | --- | --- | --- |
|  | **Frequency (cases)** | **Prevalence**  **(%)** | **Frequency (cases)** | **Prevalence (%)** |
| **0** | 172 | 57.72 | 77 | 65.25 |
| **1** | 58 | 20.14 | 22 | 18.64 |
| **2** | 22 | 7.64 | 8 | 6.78 |
| **3** | 12 | 4.17 | 4 | 3.39 |
| **No data** | 24 | 8.3 | 7 | 5.93 |
