# Supplementary material for: Identfication of viral and bacterial etiologic agents of the pertussis-like syndrome in children under 5 years old hospitalized
Source: BMC Infect Dis. 2019 Jan 21;19:75. doi: 10.1186/s12879-019-3671-6 (PMC6341522; doi:10.1186/s12879-019-3671-6)
Supplement: Supplementary file 4 — Table S4. Coinfections in hospitalized children with a probable diagnosis of Pertussis, positives for respiratory virus and atypical bacteria. (DOCX 142 kb) [file 12879_2019_3671_MOESM4_ESM.docx]

**Table S4. Clinical symptoms in hospitalized children with a probable diagnosis of Pertussis, positives for respiratory virus and atypical bacteria.**

| 1. **Clinical symptoms in hospitalized children** | | | | | | | | | | | | |
| --- | --- | --- | --- | --- | --- | --- | --- | --- | --- | --- | --- | --- |
| **Clinical**  **Symptoms** | **N= 288 (%)** | **Flu-A** | **Flu-B** | **RSV-A** | **RSV-B** | **ADV** | **PIV-1** | **PIV-2** | **PIV-3** | ***Bordetella pertussis*** | ***Mycoplasma pneumoniae*** | ***Chlamydia pneumoniae*** |
|  |  | n=6 (%) | n=57 (%) | n=43 (%) | n=5 (%) | n=141 (%) | n=5 (%) | n=2 (%) | n=2 (%) | n=118 (%) | n=75 (%) | n=51 (%) |
| Whooping | 134 (46.5) | 2 (33.3) | 18 (31.6) | 14 (32.6) | 2 (40.0) | 72 (51.1) | 1 (20.0) | 1 (50.0) | 1 (50.0) | 53 (44.9) | 38 (50.7) | 20 (39.2) |
| Cyanosis | 83 (28.8) | 2 (33.3) | 15 (26.3) | 9 (20.9) | 1 (20.0) | 40 (28.4) | 1 (20.0) | --- | 1 (50.0) | 37 (31.4) | 20 (26.7) | 10 (19.6) |
| Shortness of breath | 124 (43.1) | 2 (33.3) | 16 (28.1) | 20 (46.5) | 2 (40.0) | 61 (43.3) | 2 (40.0) | --- | 1 (50.0) | 40 (33.9) | 40 (53.3) | 19 (37.3) |
| Fever | 102 (35.4) | 2 (33.3) | 20 (35.1) | 19 (44.2) | 2 (40.0) | 54 (38.3) | 2 (40.0) | 1 (50.0) | --- | 32 (27.1) | 22 (29.3) | 17 (33.3) |
| Apnea | 47 (16.3) | 2 (33.3) | 7 (12.3) | 9 (21.0) | 2 (40.0) | 21 (14.9) | --- | --- | --- | 23 (19.5) | 7 (9.3) | 9 (17.7) |
| Diarrhea | 31 (10.8) | 1 (16.7) | 8 (14.0) | 6 (14.0) | --- | 17 (12.1) | --- | --- | --- | 14 (11.9) | 5 (6.7) | 3 (5.9) |
| Vomits | 136 (47.2) | 5 (83.3) | 19 (33.3) | 24 (55.8) | 1 (20.0) | 67 (47.5) | 3 (60.0) | 1 (50.0) | 2 (100.0) | 65 (55.1) | 31 (41.3) | 25 (49.0) |
| Stridor | 74 (25.7) | 2 (33.3) | 11 (19.3) | 8 (18.6) | --- | 38 (27.0) | 2 (40.0) | 1 (50.0) | 1 (50.0) | 32 (27.1) | 20 (26.7) | 10 (19.6) |
| Others | 16 (5.6) | --- | 5 (8.8) | 2 (4.7) | 1 (20.0) | 9 (6.4) | --- | --- | --- | 3 (2.5) | 5 (6.7) | 2 (3.9) |
| **B) Clinical symptoms in hospitalized children with a diagnostic for a single infectious agent** | | | | | | | | | | | | |
| **Clinical**  **Symptoms** | **Negatives N= 41 (%)** | **Flu-A** | **Flu-B** | **RSV-A** | **RSV-B** | **ADV** | **PIV-1** | **PIV-2** | **PIV-3** | ***Bordetella pertussis*** | ***Mycoplasma pneumoniae*** | ***Chlamydia pneumoniae*** |
|  |  | n=0 (%) | n=7 (%) | n=4 (%) | n=0 (%) | n=25 (%) | n=1 (%) | n=1 (%) | n=1 (%) | n=24 (%) | n=15 (%) | n=2 (%) |
| Paroxysm | 32 (78.0) | --- | 5 (71.4) | 3 (75.0) | --- | 22 (88.0) | 1 (100.0) | 1 (100.0) | 1 (100.0) | 23 (95.8) | 12 (80.0) | 1 (50.0) |
| Respiratory distress | 31 (75.6) | --- | 6 (85.7) | 4 (100.0) | --- | 16 (64.0) | 1 (100.0) | --- | 1 (100.0) | 21 (87.5) | 15 (100.0) | 2 (100.0) |
| Redness | 29 (70.7) | --- | 5 (71.4) | 3 (75.0) | --- | 18 (72.0) | --- | 1 (100.0) | 1 (100.0) | 23 (95.8) | 9 (60.0) | --- |
| Cyanosis | 24 (58.5) | --- | 4 (57.1) | 3 (75.0) | --- | 11 (44.0) | 1 (100.0) | 1 (100.0) | 1 (100.0) | 17 (70.8) | 4 (26.7) | 2 (100.0) |
| Breastfeeding problems | 22 (53.7) | --- | 4 (57.1) | 2 (50.0) | --- | 10 (40.0) | 1 (100.0) | --- | 1 (100.0) | 10 (41.7) | 5 (33.3) | 2 (100.0) |
| Vomits | 20 (48.8) | --- | 2 (28.6) | 3 (75.0) | --- | 6 (24.0) | --- | 1 (100.0) | 1 (100.0) | 14 (58.3) | 4 (26.7) | 1 (50.0) |
| Fever | 18 (43.9) | --- | --- | 2 (50.0) | --- | 11 (44.0) | --- | --- | --- | 10 (41.7) | 4 (26.7) | 1 (50.0) |
| Stridor | 11 (26.8) | --- | 2 (28.6) | 1 (25.0) | --- | 5 (20.0) | --- | 1 (100.0) | --- | 9 (37.5) | 3 (20.0) | --- |
| Apnea | 7 (17.1) | --- | --- | 1 (25.0) | --- | 1 (4.0) | --- | --- | --- | 9 (37.5) | 2 (13.3) | 1 (50.0) |
| Diarrhea | 4 (9.8) | --- | 1 (14.3) | --- | --- | 2 (8.0) | --- | --- | --- | 4 (16.7) | --- | 1 (50.0) |
| Others | 1 (2.4) | --- | 1 (14.3) | --- | --- | 2 (8.0) | --- | --- | --- | 1 (4.2) | 2 (13.3) | --- |
