# Supplementary material for: Identfication of viral and bacterial etiologic agents of the pertussis-like syndrome in children under 5 years old hospitalized
Source: BMC Infect Dis. 2019 Jan 21;19:75. doi: 10.1186/s12879-019-3671-6 (PMC6341522; doi:10.1186/s12879-019-3671-6)
Supplement: Supplementary file 5 — Table S5. Complications among hospitalized children with a probable diagnosis of Pertussis, positives for respiratory virus and atypical bacteria. (DOCX 123 kb) [file 12879_2019_3671_MOESM5_ESM.docx]

**Table S5. Complications among hospitalized children with a probable diagnosis of Pertussis, positives for respiratory virus and atypical bacteria.**

| **A) Complications in hospitalized children** | | | | | | | | | | | | | | |
| --- | --- | --- | --- | --- | --- | --- | --- | --- | --- | --- | --- | --- | --- | --- |
| **COMPLICATIONS** | **N= 288 (%)** | **RSV-A** | **RSV-B** | | **Flu-A** | | **Flu-B** | **ADV** | **PIV-1** | **PIV-2** | **PIV-3** | ***Bordetella pertussis*** | ***Mycoplasma pneumoniae*** | ***Chlamydia pneumoniae*** |
|  |  | n=6 (%) | n=57 (%) | | n=43 (%) | | n=5 (%) | n=141(%) | n=5(%) | n=2 (%) | n=2 (%) | n=118 (%) | n=75 (%) | n=51 (%) |
| ABOS | 163 (56.6) | 5 (83.3) | 31 (54.4) | | 26 (60.5) | | 1 (20.0) | 89 (63.1) | 1 (20.0) | --- | 2 (100) | 64 (54.2) | 38 (50.7) | 33 (64.7) |
| Pneumonia | 81 (28.1) | 1 (16.7) | 12 (21.1) | | 13 (30.2) | | 3 (60.0) | 46 (32.6) | 2 (40.0) | 1 (50.0) | --- | 25 (21.2) | 17 (22.7) | 18 (35.3) |
| Atelectasis | 33 (11.5) | 1 (16.7) | 4 (7.0) | | 9 (20.9) | | --- | 18 (12.8) | 1 (20.0) | 2 (100) | --- | 8 (6.8) | 9 (12.0) | 10 (19.6) |
| Seizures | 3 (1.0) | --- | 1 (1.8) | | --- | | --- | 2 (1.4) | --- | --- | --- | 1 (0.9) | 2 (2.7) | 0 (0.0) |
| Umbilical hernia | 7 (2.4) | 1 (16.7) | 3 (5.3) | | 1 (2.3) | | --- | 5 (3.6) | 1 (20.0) | --- | --- | 6 (5.1) | 1 (1.3) | 2 (3.9) |
| Others | 14 (4.9) | --- | 1 (1.8) | | 4 (9.3) | | --- | 6 (4.3) | --- | --- | --- | 5 (4.2) | 7 (9.3) | 4 (7.8) |
| **B) Complications in hospitalized children with a diagnostic for a single infectious agent** | | | | | | | | | | | | | | |
| **COMPLICATIONS** | **NegativesN= 41(%)** | **Flu-A** | | **Flu-B** | **RSV-A** | **RSV-B** | | **ADV** | **PIV-1** | **PIV-2** | **PIV-3** | ***Bordetella pertussis*** | ***Mycoplasma pneumoniae*** | ***Chlamydia pneumoniae*** |
|  |  | n=0 (%) | | n=7 (%) | n=4 (%) | n=0 (%) | | n=25 (%) | n=1 (%) | n=1 (%) | n=1 (%) | n=24 (%) | n=15 (%) | n=2 (%) |
| ABOS | 25 (61.0) | **---** | | 5 (71.4) | 2 (50.0) | --- | | 13 (52.0) | --- | --- | 1 (100.0) | 11 (45.8) | 8 (53.3) | 1 (50.0) |
| Pneumonia | 17 (41.5) | **---** | | 1 (14.3) | --- | **---** | | 7 (28.0) | 1 (100) | 1 (100) | --- | 3 (12.5) | 1 (6.7) | 1 (50.0) |
| Atelectasis | 6 (14.6) | **---** | | --- | --- | ***---*** | | 2 (8.0) | 1 (100) | 1 (100) | --- | --- | --- | 1 (50.0) |
| Seizures | 1 (2.4) | **---** | | --- | --- | ***---*** | | --- | --- | --- | --- | --- | --- | --- |
| Umbilical hernia | --- | **---** | | --- | --- | ***---*** | | --- | 1 (100) | --- | --- | --- | --- | --- |
| Others | 2 (4.9) | **---** | | --- | --- | ***---*** | | --- | --- | --- | --- | 1 (4.2) | 1 (6.7) | --- |
